# An evaluation of a community-based food supplementation for people living with HIV in Ghana: implications for community-based interventions in Ghana

**DOI:** 10.1186/s13104-015-1511-3

**Published:** 2015-10-01

**Authors:** Kofi Akohene Mensah, Paul Okyere, Paul Narh Doku

**Affiliations:** School of Public Health, Kwame Nkrumah University of Science and Technology, Kumasi, Ghana; Department of Psychology, University of Ghana, Accra, Ghana

**Keywords:** Community-based care, HIV/AIDS, Food supplementation, Weight gained, Support groups, Stakeholders, Programme evaluation

## Abstract

**Background:**

Community-based care and support services are limited in sub-Saharan Africa and as a result a high number of people living with HIV (PLHIV) are not getting the required care and support services. The aim of this study was to assess the impact of food supplementation services for PLHIV in Ghana on weight gained and factors associated with weight gained.

**Methods:**

The study employed mixed methods study design involving quantitative and qualitative techniques. These were structured questionnaire administered to 200 PLHIV selected through simple random sampling and a qualitative component consisting of 14 semi-structured interviews with purposefully selected stakeholders and eight focus group discussions with the beneficiaries.

**Results:**

The analysis of the quantitative data showed on average, beneficiaries had gained weight [mean difference in weight was 2 kg with 95 % CI (1.1, 2.9), *P* value <0.001]. Multivariate analysis showed that the support group to which the beneficiary belonged was the most important determinant of gaining weight. Through the qualitative interviews, beneficiaries indicated that the anti-retroviral drugs were making them hungry and the food helped to alleviate that effect. Notwithstanding, they indicated that the food was nutritious, made them healthy and strong, contributed to their weight gain and was their main sources of hope at home when they had no money.

**Conclusions:**

A broad strategy of food supplementation for PLHIV should be implemented in different ways for different support groups taking into account the differences between different support groups when planning such an intervention.

**Electronic supplementary material:**

The online version of this article (doi:10.1186/s13104-015-1511-3) contains supplementary material, which is available to authorized users.

## Background

The devastating effect of HIV/AIDS on sub-Saharan Africa is overwhelming. By the end of 2008, an estimated 22.4 million people were living with HIV and approximately 1.9 million additional people were infected with HIV during that year [1]. In Africa, food shortages and malnutrition have combined with the HIV/AIDS pandemic to bring many countries to the edge of a crisis [2]. The relationship between HIV/AIDS and malnutrition cannot be underestimated [3–5]. Malnutrition can increase the risk of HIV transmission from mothers to babies and the progression of the infection. This relationship was observed in a study conducted in three African countries (Tanzania, Zambia and Malawi) which established that anaemia, poor weight gain and low BMI in HIV-infected pregnant women were significantly associated with mother-to-child transmission of HIV [6]. In turn, HIV infection can affect malnutrition through attacking the immune system, which ultimately affects the nutrient intake of the affected [7, 8]. However, with appropriate nutritional intervention, people living with HIV (PLHIV) can build their reserves by meeting their energy and protein requirements, decreasing their vulnerability to weight loss and wasting that accompany diarrhoea and other opportunistic infections [9].

In Ghana, 300,000 people are estimated to be living with HIV/AIDS and only 70,000 of them are reported to be having access to antiretroviral therapy (ARVs) because they are scarce and expensive [10]. The Ghana AIDS Commission and United States Agency for International Development (USAID) Ghana strategic plan, both indicate that care and support services for PLHIV and orphans are grossly inadequate and that the institutional capacity, training, resources, food and cash to provide these services by the public and private sectors are limited and sometimes non-existent. A baseline study conducted by Opportunity Industrialisation Centre International (2003) also indicated that PLHIV in Ghana sought treatment from traditional healers and elderly family members back in their villages where most often inadequate food supply and malnutrition are major problems [11].

## Theoretical framework

The relationship between HIV/AIDS and food insecurity in sub-Saharan Africa have been characterized as a syndemic not only because they coexist but also they are both embedded in social inequalities and the presence of one condition is likely to compound the other [12, 13]. Syndemic theory posits that an understanding of the HIV/AIDS pandemic can adequately be understood by examining the complex interaction of the disease with other health problems such as malnutrition, tuberculosis, sexually transmitted infections in the context of social and structural conditions such as poverty and social inequalities [14]. Using syndemic theory, we now understand the complex interactions between HIV infection, AIDS illness and malnutrition which exacerbate health outcomes for millions of people in sub-Saharan Africa. On one hand HIV/AIDS can have debilitating effect on abilities of households and families to provide for themselves. For example, HIV may weaken the immune system of the affected and hamper his/her ability to work productively [15]. And on the other hand, individuals, particularly women who are living in food insecure households may be compelled to engage in risky economic ventures such as unsafe sexual practices which may increase their risk of acquiring HIV [16]. Clearly, there is a complex interaction between HIV/AIDS and food insecurity and malnutrition. The theory of syndemic provides a useful framework to understand the mutually reinforcing interrelationships between HIV/AIDS and food insecurity and malnutrition which combine to produce poor health outcomes. In view of this, programmes that are likely to be effective must be multi-level and multi-dimensional in nature that recognises the complex intersection of HIV/AIDS, food insecurity and malnutrition.

## Background information about the HOPE programme

Opportunity Industrialization Centre International (OICI) Ghana in partnership and with funding from the United States Agency for International Development (USAID/Ghana), implemented a 5 year food insecurity reduction programme called ENHANCE (enhancement of household agriculture, nutrition, risks reduction and community empowerment). ENHANCE had a special HIV/AIDS programme component, named HOPE (HIV/AIDS Orphans and Vulnerable Children and PLHIV Care, support and Economic Opportunities Enhancement Programme). The goal of the HOPE programme was to improve care, support and economic opportunities for Orphans and Vulnerable Children (OVCs) and PLHIV in high HIV/AIDS prevalence areas—Ashanti, Eastern, Greater Accra and Western Regions—from 2004 to 2009. It was a 5 year programme which began in 2004 and ended in 2009. OICI Ghana linked up with the network of HIV/AIDS associations which were on the ground to identify and select PLHIV groups benefiting from the programme.

The interventions included the provision of monthly dry micro-nutrient dense food rations composed of Soy Fortified Wheat and Vegetable Oil to PLHIV and OVC. In addition, the programme sought to build capacity and increase the knowledge and skills of the beneficiaries through vocational skills, entrepreneurial, business development, monthly health education and training workshops. The aim was to sustain livelihoods, promote behaviour change and communicate information. The intended outcomes were improved health, disease prevention and the alleviation of severe malnutrition. The programme started in February 2004 with 400 beneficiaries and as at the time of this study had over 1000 beneficiaries. The beneficiaries who enrolled on the Hope programme were assigned to one of 21 support groups. The support groups were the direct beneficiaries of the HOPE programme and they were PLHIV in the four operational regions of the programme. The support groups were run by local volunteers with the help of the HOPE programme staff. Funding for the interventions for the support group came from the USAID with a small amount (0.5 %) from the Ghana AIDS Commission.

This study reports on the impact of the food supplementation component of the programme, to provide lessons on weight gained and factors associated with gaining weight. It further presents the views of the patients and stakeholders on the food and its sustainability.

## Methods

### Quantitative component

#### Study setting

This was a multisite study involving four (4) out of ten (10) administrative regions of Ghana namely Greater Accra, Ashanti, Western and Eastern regions. These were the four operational areas of the HOPE programme. Greater Accra is the most densely populated and urbanised region of Ghana. According to the 2010 population and housing census, almost 90 % of the region’s population have received some form of formal education [17]. In terms of economic activities, majority of the economically active population (15 years and older) are involved in wholesale and retail trading although the region boasts of the highest proportion of professionals compared to all the other regions. Ashanti region is the most populous region in Ghana and the second most urbanised in Ghana after Greater Accra. In terms of economic activities most of the inhabitants are into agriculture (44.5 %) with the remainder involved in trading (18.4 %), manufacturing (12.2 %) and social and personal services (9.9 %). About 15.4 % of the region’s population (6 years and older) have never been to school. Eastern and Western regions have similar social, economic and cultural characteristics similar to Ashanti region.

#### Instrumentation

A structured questionnaire was administered to 200 PLHIV who were recruited through a simple random sampling technique. This sampling strategy was used to select the PLHIV from the 21 support groups. The sampling strategy created a list of substitutes to be approached in a similar way to take the place of individuals who declined to be part of the study. As a result, a total number of 200 PLHIV were sampled at regular intervals from the sampling frame which was 997. PLHIV were numbered from 1 to 997 based on the support group lists supplied by the ‘HOPE’ director. The 200 PLHIV were selected from a table of random numbers [18]. Since the sampling frame consisted of 997 units, three digits were used to ensure that all 997 had an equal chance of being included. Without looking at the table, a pen was used to pinpoint one number on the row and two numbers on the column and moved down the page to select the required numbers within the range of 1–997. On moving down the page, numbers within the range of 1–997 were taken. If this was not possible, then movement continued in the same direction until a number within that range was found. This continued until a total number of 200 were selected (Additional file 1).

The names of the selected 200 PLHIV were ticked in the support groups list. The first 200 PLHIV bearing those numbers were approached at their support group meetings and engaged in structured interviews with their consent. The data gathered from the PLHIV included information about their background, current circumstances and the interventions they have experienced. As at the time of the study, none of the participants had dropout of the programme. The data was collected at the end of the programme in 2009 and this study is the outcome of that evaluation.

#### Anthropometry measurements

The anthropometric measurements were undertaken by the first author with the help of two trained research assistants. The first author and research assistants who measured the body weight and height were trained on the instruments used for both measurements (Salter scale for body weight measurement and body height metre for height measurement). The researchers practiced among themselves to minimise any potential mistakes. Also, the two trained research assistants were monitored and supervised whilst taking the measurements. The weight of all the beneficiaries was taken before they were enrolled on the HOPE programme. In the course of the study, the current weights of the beneficiaries were taken and compared with the previous/baseline weight. This enabled the researchers to ascertain whether beneficiaries maintained, gained or lost weight.

### Statistical analysis

The null hypothesis of no difference in previous and current weight was tested using a paired sample t test. A logistic regression was further performed using maintaining and gaining weight versus losing weight as the dependent variable and support group, age group, sex, previous occupation, current occupation, highest level of education, household size, number of children, length of time on the programme, ART status, entrepreneurial skills, number of skills received and number using as a vocation as independent variables. Any independent variable that had a significant association with the outcome went forward into a multiple logistic regression model which also included age and sex. The data was analysed using SPSS version 15.0.

### Qualitative methods

The qualitative methods employed were eight focus group discussions with beneficiaries and 14 interviews with stakeholders. The focus groups were sampled from the 21 support groups using a simple random technique. In this case, twenty-one pieces of paper bearing the names of the 21 support groups were folded and placed in a bowl. This was shaken and the first eight support groups selected were contacted for the focus group discussions. The second part of the randomisation process focussed on selection of participants for the focus group. This resulted in each of the selected support group having eight PLHIV using a paper ballot. Each group consisted of eight PLHIV and groups were facilitated by the first author. The focus group discussions were held at the support groups meeting room. The focus group schedule asked about the support received from the programme and its contribution to their health and wellbeing, any difficulties or challenges encountered and any recommendations for changes to the programme.

Stakeholders directly involved in the programme implementation were approached directly and their participation sought. The stakeholders were selected purposefully. The content of the study was explained to them using an information sheet. They were then asked to sign consent forms indicating their agreement to participate in the study. The sample included eight support group coordinators, the four OICI regional coordinators, the OICI Monitoring and Evaluation Officer and the National HIV/AIDS Director of OICI Ghana ‘HOPE’ programme. The support group coordinators selected were those in-charges of the support groups selected for focus discussions in order that any emerging issues from the focus group discussions could be further explored. Interviews with the stakeholders gathered information on the structure of the programme, the core activities, how well the programme has progressed in accordance with the goals and objectives of the programme, challenges and recommendations for the future.

All the focus group discussions and the interviews were audio recorded and additional notes were taken by the researcher where necessary. The audio recordings of focus group discussions and semi-structured interviews were transcribed verbatim. The data was analysed using thematic analysis [19] facilitated by software (ATLAS Ti). Initial codes were applied to the transcripts, refined and subsequently sorted into potential themes. These were then grouped into broad categorical headings, which both map onto the questions asked. These were: benefits of the programme, constraints and suggested policy or broad changes.

### Ethical consideration

Ethical approval was sought and granted from the University of Glasgow and School of Medical Sciences (Committee on Human Research, Publication and Ethics) in the United Kingdom and Ghana, respectively. Participants were informed that their responses would be kept confidential and informed consent was obtained from participants.

## Results

### Quantitative component

#### Profile of study participants

The profile of the respondents is described in Table 1 in Appendix. The mean age of the respondents was 39.9 years (SD = 9.4) with 39.0 % falling within the age group 30–39 years. Females represented 82.0 % of the respondents. Out of the 200 respondents, 77 (38.5 %) had stayed on the programme for less than 12 months, 65 (32.5 %) had stayed on for 12–24 months and the rest (29.0 %) had stayed more than 24 months. Seventy-seven percent were on Antiretroviral drugs (ARVs), while 23 % were not.

**Table 1:** Profile of study participants

Variable	Frequency (n = 200)	Percentage (%)
Age group (years)		
<30	26	13.0
30–39	78	39.0
40–49	63	31.5
50–59	26	13.0
≥60	7	3.5
Mean = 39.9; SD = 9.4		
Previous occupation		
Farmer	34	17.0
Trader	104	52.5
Unemployed	17	8.5
Others	44	22.0
Current occupation		
Farmer	24	12.0
Trader	71	35.5
Unemployed	75	37.5
Others	30	15.0
Education		
Basic	135	67.5
Secondary	13	6.5
Tertiary	10	5.0
None	42	21.0
Length of time on the programme		
<12 months	77	38.5
12–24 months	65	32.5
>24 months	58	29.0

### Weight gained and factors associated with gaining/maintaining weight

The analysis of the quantitative data showed that beneficiaries experienced a positive outcome from the nutritional interventions. Almost two-thirds (64.5 %) of the respondents gained weight whilst 30.5 % of the respondents lost weight (their weights at enrolment into the programme had dropped as at the time of the study) and 5.0 % maintained weight (their weight remained the same from baseline to the time of the study). The mean previous/baseline weight (the recorded weight on enrolment in the HOPE programme) was 58.0 kg (SD = 11.7 kg). The weight at the time of the study (referred to as current weight) had a mean of 60.0 kg (SD = 11.9 kg). The mean difference in weight was 2 kg with 95 % CI (1.1, 2.9), p value <0.001. The mean differences in weight of those on ART and those not on ART was 1.9 and 2.3 kg, respectively. Multivariate analysis showed that the support group to which the beneficiary belonged was the most important determinant of a positive outcome as shown in Table 2 in Appendix (p value = 0.002). There were no statistically significant differences between age group, sex, ART status, length of time on the programme, current occupation and the odds of maintaining/gaining weight after adjustment. It is also important to note that the odds of maintaining/gaining weight for PLHIV on ART were less than half as compared to those not on ART. These results were significant at the 10 % level (p value = 0.1).

**Table 2:** Multiple regression of maintaining/gaining weight vs. losing weight (N = 200)

Variable	OR 95 % CI	P value
Overall effect of support group	1	0.002
Overall effect of age group	1	0.6
Overall effect of highest level of education	1	0.3
Overall effect of length of time on the programme	1	0.2
Overall effect of ART status	1	0.1

### Qualitative results

#### Beneficiaries and stakeholders view on the food supplementation

Findings from the interviews and focus group discussions demonstrated that the programme was perceived as beneficial to health and nutritional status:

Before they came to help us the disease was killing us massively, we were dying of the disease, but since they came in, the rate has reduced (Participant, FG 2).

[…] the food helps us a lot. It gives us strength and when we check our body weight, we observe that we have gained some weight (Participant, FG 4).

They reported that the anti-retroviral drugs were making them hungry and the food has contributed tremendously to alleviate that effect:

When you are put on ARV it is another thing all together. You get hungry, you need to eat and you need to take your drugs. How do you take your drug when you don’t have food? It is a challenge, so most people were not adhering. They tell us that, I am not taking my drugs because when I take my drug I get hungry and I don’t have money to buy food. So adherence to ARV increased rather than decreased (Stakeholder Participant).

Additionally, they reported that support groups have been sustained, membership had increased and most of the PLHIV attended meetings because of the food:

If there is food, the association will be sustained or we will get more people to join but if they refused to give us food, some of them won’t come but go and join other associations (Participant, FG 4).

You realise that food, although is phasing out but it has always been able to always bring these people together so that if you have any other thing that you want to tell them, you can tell them but without the food normally you see that they do not even attend their monthly meetings, so if you go there for education, no matter the kind of education you coming with, all they are interested is the food (Stakeholder Participant).

In addition to the food supplement, beneficiaries were taken through training and workshops. Beneficiaries indicated receiving education around drug adherence; nutritional advice; personal hygiene and life style; and HIV/AIDS and its side effects. They reported that this provided them with hope and motivation:

They gave us hope and motivated us not to isolate ourselves or look down on ourselves because of our situation. So they always encourage us to be happy so that we would not be sad about our situation (Participant, FG 8).

As a result of your help and education we’ve put away all anxieties and have taken the path of protecting ourselves and living a better life (Participant, FG 3).

Nonetheless, there were challenges that hampered the smooth implementation of the programme. Beneficiaries reported that the quantity of food received was reduced in the course of programme implementation to give equal quantities to all groups receiving support from the USAID in order to control participants moving from one support group to another.

In addition, some indicated that food should be varied for them since they were fed up eating one type of meal. Also, the long term sustainability of the food supplementation once funding is discontinued was a concern due to the non-local nature of the food supplements. Finally, they mentioned selling some of the food to earn money to pay for their medications:

The situation is, eating the food is very helpful but due to financial difficulties some of us sell the food. The reasons we do that is that, sometimes we need to buy the drugs meanwhile you are financially handicap, so you are forced to do so even though that is not desired (Participant, FG 3).

Most of them depend on the food to take their drugs and I don’t know where some of them their daily bread will come from when the programme ends since most of them cannot afford to sustain the food (Stakeholder Participant).

## Discussion

Beneficiaries and stakeholders were highly satisfied with the food support as indicated in the discussions and the interviews. The high level of satisfaction of the food might have contributed to improvement in the current weight as compared to the previous weight since weight is gained by eating a variety of food more frequently [20]. Additionally, a range of factors were associated with maintaining and gaining weight. However, only support groups proved to be highly significant after adjustment. The likely explanation is that some Support Groups were more successful in creating an atmosphere of hope and positively allied to their success in generating community based social networks (Support Groups) [21]. In this study, beneficiaries confirmed that the support group had generated hope and motivated them to live positively. However, belonging to certain support groups were highly associated with maintaining and gaining weight as compared to others.

This suggests that the difference in care provided varied from one support group to other. For example, Support Group 9 had the best outcome in terms of weight. It had been providing its members with food supplementation before the commencement of the programme which suggests that the outcomes recorded in this study reflect a longer period of sustained inputs. By way of contrast, Support Group 10 recorded the poorest weight outcomes. The most likely explanation, based on interview data, is the frequent delays in food provision and questions about food quality. The fundamental problem seemed to have been a slow programme response to increasing membership of this support group. As a result, food was being overstretched to feed the entire membership which obviously compromised the distribution formula.

In spite of the above, the mean difference in weight of 2 kg for this study was relatively higher than that recorded in a similar study in Uganda where the significant mean difference in weight was 0.36 kg [22]. Even though, the difference in weight for this study was based on varying periods of 60 months whilst that of Uganda was for 12 months, multivariable analysis for this study indicated that the length of time on the programme is not associated with gaining or maintaining weight. It is possible that the mean difference in weight might have been higher than what was observed if the initial quantity of food had not been reduced and had food not been sold. Also, the Uganda study indicated no significant association with ART and gaining weight which is further confirmed in this study where after statistical adjustment, those on ART were almost 60 % less likely to maintain or gain weight (p value = 0.1). However, the food supplementation contributed to improve ART acceptance and adherence as indicated by the beneficiaries and the stakeholders. Food supplementation has been established to increase ART adherence: this was confirmed in a randomised study in Zambia where patients on antiretroviral therapy and received food supplementation improved ART adherence by 40 % as compared to patients on antiretroviral therapy alone without food supplements [23].

Finally, the types of food provided by the programme brought tremendous benefits to the beneficiaries (as indicated in the focus group discussion and the interview). However, the sustainability of the food component beyond the programme period remains a major concern. Part of the reason might be that the foods are not locally produced and beneficiaries had developed a taste for these imported foods. This in itself is not so much of a problem but switching over to locally available and produced foods may be a sensible policy for the future. It seems logical to suggest that the programme should have invested in the production of locally available foods for the beneficiaries instead of importing the foods which are not sustainable beyond the programme period. The investment in local production through community farming will mean that beneficiaries could enjoy the produce during and after the implementation of the programme. In addition, it would have given employment to beneficiaries interested in farming. The use of locally available foods together with effective approaches has been identified to ensure programme sustainability in other studies. This approach was used in Vietnam to promote appropriate infant feeding practices and 3 years after the programme implementation, mothers were still practising with the new behaviour acquired from the programme [24].

This study also provides lessons for the interrelationships between HIV/AIDS and food security in sub-Saharan Africa. The impact of HIV/AIDS on food insecurity is well-documented in Africa as it negatively affects household productive capacity, decreases household agricultural output, and reduces income for food purchases [25, 26]. On the other hand, food insecurity has been shown to worsen the vulnerability of PLHIV through increased behavioural risk of HIV transmission, reduced access to HIV treatment and care and decreased ART adherence [27]. There is therefore the need to strengthen institutional capacity in sub-Saharan Africa and assist households affected by HIV/AIDS to maintain food production and security and mitigate the vulnerability to this pandemic.

## Strengths and limitations

The multiple approaches employed enabled the study to be more comprehensive which facilitated the drawing together of both qualitative and quantitative findings which in turn, made the conclusions more meaningful and robust. Furthermore, the study provides unique information on community based care and support for people living with HIV in Ghana and further provides lesson learning for Ghana, Africa and other parts of the developing world. This strength is only such because of the very limited published information on care and support for people living with HIV in Ghana and Africa generally. Therefore, the findings will help in the design of future programme as well as improving existing programmes.

On the other hand because the study commenced at a late stage of the programme implementation it was not possible to identify non-interventional groups to compare the findings from programme recipients. Therefore, the findings could not be attributed entirely to the intervention. It would have been better if it had been possible to carry out a prospective evaluative study using cluster randomised controlled trial methods in order to attribute causality to the intervention. However, such an approach would only have been possible if decisions leading to a trial had been made prior to the programme implementation and, even if such decisions had been made, the problem of identifying a suitable control group and the ethics of creating a control group would have been insurmountable.

## Conclusions

The study shows that participants gained weight. A wide range of factors were associated with gaining weight (support group, sex, farmers, ART status, length of time on the programme and marital status) but the centre that provided care had a strong and independent effect. This suggests that the way in which care was provided varied from centre to centre and some were more successful than others. The theoretical framework of syndemic theory serves as a useful guide in understanding the intersection of HIV/AIDS, food shortages and malnutrition. These interrelated factors mutually reinforce each other and work together produce adverse health outcomes people with HIV/AIDS. Finally, our study has revealed that a broad strategy of food supplementation for PLHIV will inevitably be implemented in different ways by different support groups, and these differences must be recognised and taken into accounts in planning of such an intervention.

## Additional file

10.1186/s13104-015-1511-3 Sample questionnaire instrument.

## Appendix

See Tables 1, 2.

## References

[1] UNAIDS/WHO. Aids epidemic update: December. 2009. http://data.unaids.org/pub/Report/2009/JC1700_Epi_Update_2009_en.pdf. Accessed 10 Jan 2015.

[2] World Health Organization (2005). Consultation on nutrition and HIV/AIDS in Africa: evidence, lessons and recommendations for action.

[3] Nnyepi MS (2009). The risk of developing malnutrition in people living with HIV/AIDS: observations from six support groups in Botswana. South Afr J Clin Nutr.

[4] Mahlungulu S, Grobler LA, Visser ME, Volmink J. Nutritional interventions for reducing morbidity and mortality in people with HIV. Cochrane Database Syst Rev. 2007;3. doi:10.1002/14651858.CD004536.pub2. 10.1002/14651858.CD004536.pub2 17636766

[5] Piwoz E, Bonnard P, Castlema T, Cogill B, Elder L, Remancus S, Tanner C. Nutrition and HIV/AIDS: evidence, gaps, and priority actions. 2004. http://pdf.usaid.gov/pdf_docs/Pnacy055.pdf. Accessed 14 Dec 2014.

[6] Mehta S, Manji KP, Young AM, Brown ER, Chasela C, Taha TE, Read JS, Goldenberg RL, Fawzi WW (2008). Nutritional indicators of adverse pregnancy outcomes and mother-to-child transmission of HIV among HIV-infected women. Am J Clin Nutr.

[7] Duggal S, Das Chugh T, Duggal AK (2012). HIV and malnutrition: effects on immune system. Clin Develop Immunol.

[8] Castlemen T, Seumo-Fosso E, Cogill B (2003). Food and nutrition implications of antiretroviral therapy in resource limited settings (Technical Note No 7).

[9] World Health Organization. Aids treatment, nutrition and food supplements. 2005. http://www.who.int/3by5/mediacentre/fsFood/en/. Accessed 28 Feb 2015.

[10] Okine V. Only 10 percent of Ghanaians know their HIV status. 2010. http://www.ghanaweb.com/GhanaHomePage/NewsArchive/artikel.php?ID=180497. Accessed 15 Dec 2014.

[11] Opportunities Industrialisation Centre International. Baseline survey report. Unpublished internal report. Accra, Ghana: OICI; 2003.

[12] Himmelgreen D, Romero-Daza N, Turkon D, Watson S, Okello-Uma I, Sellen D (2009). Addressing the HIV/AIDS-food insecurity syndemic in sub-Saharan Africa. Afr J AIDS Res.

[13] Garcia J, Hromi-Fiedler A, Mazur RE, Marquis G, Sellen D, Lartey A, Perez-Escamilla R (2013). Persistent household food insecurity, HIV, and maternal stress in peri-urban Ghana. BMC Public Health.

[14] Klein H (2011). Using a syndemic theory approach to studying HIV risk taking in a population of men who use the internet to find partners for unprotected sex. Am J Men’s Health.

[15] Singer M (2011). Toward a critical biosocial model of ecohealth in Southern Africa: the HIV/AIDS and nutrition insecurity syndemic. Ann Anthropol Prac.

[16] Weiser S, Leiter K, Bangsberg DR, Butlers LM, Percy de Korte F, Hlanze Z, Phaladze N, Iacopino V, Heisler M (2007). Food insufficiency is associated with high-risk sexual behavior among women in Botswana and Swaziland. PLoS Med.

[17] Ghana Statistical Service (2012). 2010 population and housing census: summary report of final results.

[18] Varkevisser C, Pathmanathan I, Brownlee A. Designing and conducting health system research projects, vol. I, p. 212. Amsterdam: KIT Publishers ad IDRC; 1991.

[19] Braun V, Clarke V (2006). Using thematic analysis in psychology. Qual Res Psychol.

[20] Whitney EN, Hamilton EM, Rolfes S (1990). Understanding nutrition.

[21] Harris GE (2006). Practicing HIV/AIDS community-based research. AIDS Care.

[22] Rawat R, Kadiyala S, McNamara P. The impact of food assistance on weight gain and disease progression among HIV-infected individuals accessing AIDS care and treatment services in Uganda. BMC Public Health. 2010;316–24. 10.1186/1471-2458-10-316 PMC2896357 20529283

[23] Cantrell RA, Sinkala M, Megazinni K, Lawson-Marriott S, Washington S, Chi BH, Tambatamba-Chapula B, Levy J, Stringer EM, Mulenga L, Stringer JS (2008). A pilot study of food supplementation to improve adherence to antiretroviral therapy among food-insecure adults in Lusaka, Zambia. J Acquir Immune Defic Syndr.

[24] Marsh D, Schroeder D, Dearden K, Sternin J, Sternin M (2004). The power of positive deviance. Br Med J.

[25] Muzari W, Mpofu A, Musiyandak S (2014). Gatsi: The impacts of the HIV/AIDS pandemic on agriculture, food security and rural livelihoods in Zimbabwe. Int J Sci.

[26] Bukusuba J, Kikafunda JK, Whitehead RG (2007). Food security status in households of people living with HIV/AIDS (PLWHA). Br J Nutr.

[27] Weiser SD, Fongillo EA, Ragland K, Hogg RS, Riley ED, Bangsberg DR (2009). Food insecurity is associated with incomplete HIV RNA suppression among homeless and marginally housed HIV-infected individuals in San Francisco. J Gen Intern Med.

