# Supplementary material for: An evaluation of a community-based food supplementation for people living with HIV in Ghana: implications for community-based interventions in Ghana
Source: BMC Res Notes. 2015 Oct 1;8:519. doi: 10.1186/s13104-015-1511-3 (PMC4590264; doi:10.1186/s13104-015-1511-3)
Supplement: Supplementary file 1 — 10.1186/s13104-015-1511-3 Sample questionnaire instrument. [file 13104_2015_1511_MOESM1_ESM.docx]

**QUESTIONNAIRE**

**Study Topic: *An evaluation of a community-based food supplementation for people living with HIV in Ghana: Implications for community-based interventions in Ghana***

**An Interview based questionnaire for the evaluation of OICI Ghana HOPE**

**Project for PLWHAS in Ghana**

**Date:………………… Interviewer:……………….. Support Group Code:…...**

**Location: ………….**

**Section A: Household demographic characteristics:**

A1. Individual Code #………………. A2.Region ………………….

A3.District ……………….. A4. Town/village………………..

A5. House # ……………. A6. Sex Male [ ] Female [ ]

A7. Religion

Christian [ ] Moslem [ ] Others [ ]

A8. Ethnic group

Akan [ ] Ewe [ ] GA [ ] Fante [ ] Northerner [ ] Others [ ]

A9. Previous Occupation

Artisan [ ] Farmer [ ] Trader [ ] Hairdresser [ ] Student [ ] Unemployed [ ] Others [ ]

A10. Current occupation

Artisan [ ] Farmer Trade [ ] Hairdresser [ ] Student [ ] Unemployed [ ]

Others [ ]

A11.Highest level of Education

None [ ] Koranica [ ] Primary [ ] JSS [ ] SSS [ ] College [ ] University [ ] Polytechnic [ ]

A12. Marital status

Single [ ] Married [ ] Divorced / Separated [ ] Widowed / widower [ ] others [ ]

A13. Household size

Less than 2 [ ] 2-3 [ ] 4-5 [ ] 6-7 [ ] Above 7 [ ]

A.14. Number of children

0 [ ] 1-2 [ ] 3-4 [ ] 5-6 [ ] Above 6 [ ]

**Section B: Height Measurements:**

B1. Height 1 (m)…………………… Previous Weight ……………………………..

B2. Current Weight ……………………………

**Section C: Food Distributions and utilization**

C1. What are the various dry food rations you receive monthly?

| No | Name of Dry Ration | Quantity Received |
| --- | --- | --- |
| 1 |  |  |
| 2 |  |  |
| 3 |  |  |

C2. How long have you received the food support from OICI Ghana Project?

Less than 6 months [ ] 6-12 months [ ] 12-24 months [ ] Above 24 Months [ ]

C3. What do you do with the foods given?

Eat All [ ] Eat and sell some [ ] Sell All [ ] Give it out as a gift [ ]

Eat and give some out as a gift [ ] Sell and give out some as a gift [ ]

C4. What meals do you prepare from the foods received?

| No. | Name of Food Ration | Types of Meals | No. of individuals consumed |
| --- | --- | --- | --- |
| 1. |  |  |  |
| 2. |  |  |  |
| 3. |  |  |  |
| 4. |  |  |  |

C5. Can eating of any of the food given cause you or your family to become ill?

Yes [ ] No [ ]

If yes, please name the type of ration or food and the sickness;

| N. | Food Ration | Sickness |
| --- | --- | --- |
| 1. |  |  |
| 2. |  |  |
| 3. |  |  |

C6. Do you like all the food Rations given?

Yes [ ] No [ ]

If No, state the one of less interest and the reasons.

C7. Has the consumption of the food brought any improvement in your wellbeing or changes to you?

The same [ ] Decreased [ ] Increased [ ]

C8. What are those changes?

C9. Are you on antiretroviral therapy?

Yes [ ] No [ ]

C10. If No, why?

No money [ ] not heard of ART [ ] not interested [ ] Not due for ART [ ]

Not provided locally [ ]

**Section D: Entrepreneurial skills**

D1. Name the various entrepreneurial skills you have received from OICI

Soap making [ ] Powder preparation [ ] Yoghurt preparation [ ]

Jam preparations [ ] none [ ] others [ ]

D2. How many of the entrepreneurial skills have you received training?

None [ ] 1 [ ] 2 [ ] 3 [ ] 4 [ ] above 4 [ ]

D3. Were you involved in the selection of the entrepreneurial skills?

Yes [ ] No [ ]

D4. Are you using any of them as a vocation?

Yes [ ] No [ ]

If yes go to D 6

D5. If, No, why?

D6. Are you happy to use those entrepreneurial skills as a vocation?

Yes [ ] No [ ]

If yes, go to D8

D7. If No, why?

D8. What skills would you have preferred most if you were given the chance to do so?
